# Supplementary material for: SyNDI: synchronous network data integration framework
Source: BMC Bioinformatics. 2018 Nov 6;19:403. doi: 10.1186/s12859-018-2426-5 (PMC6219086; doi:10.1186/s12859-018-2426-5)
Supplement: Supplementary file 5 — Files of the example presented in Additional file 4. This additional file is a zip package that contains all files used in Additional file 4. Detailed descriptions of each of these files are presented in a README file included in this zip package. (GZ 30411 kb) [file 12859_2018_2426_MOESM5_ESM.gz › SyNDITools/Meme2Fimo/meme_4.9.0_4.tar.gz!/meme_4.9.0/doc/mhmme.xml]

mhmme


# mhmme -- not currently supported

**Usage:** mhmme [options] <*HMM filename*>

**Description:**

Given a Meta-MEME hidden Markov model (HMM),
`mhmme` randomly generates sequence from the model and
prints them in Fasta format.

**Input:**

- <*HMM file*> - An HMM in Meta-MEME format must be
  provided. If the filename is given as '-', then 'mhmme' will
  attempt to read the HMM from standard input.

**Output:**

The program prints to standard output one or more
sequences in Fasta format.

**Options:**

- `--numseq <integer>` The number of
  sequences to emit. Default is 1.
- `--occurs` Rather than printing the raw sequence,
  print a pseudo-sequence in which non-motif regions are represented
  as hyphens and motifs are represented like this:
  `*____7___*`, where `7` is the motif
  index.
- `--seed <integer>` Set the seed for the
  random number generator. By default, the seed is set from the
  clock.

**Bugs:** None known.

Author: William
Stafford Noble.
